# Recovery of cardiogenic shock with ablation of incessant tachycardia in a neonate

**DOI:** 10.21542/gcsp.2022.3

**Published:** 2022-06-30

**Authors:** Omnia Kamel, Tarek Hammouda, Aliaa Tarek, Wessam Ali

**Affiliations:** 1 Aswan Heart Centre, Magdi Yacoub Foundation, Aswan, Egypt

## Abstract

Supraventricular tachycardia (SVT) is one of the most common conditions in neonates that require emergency cardiac care. Its incidence in infancy is 0.06 and 0.25 per 1000 patients per year by the age of 1 month and one year respectively.

The symptoms are usually nonspecific and include poor feeding, irritability, vomiting, cyanosis, and pallid spells. If the symptoms are unrecognized for hours to days, the infant can present with significant hemodynamic compromise or heart failure. Despite the success of conservative management in most cases, catheter ablation is required in cases of failure of medical treatment.

We report a case of SVT ablation using a single catheter in a neonate who presented with tachycardia-inducedcardiomyopathy (TIC).

## Case report

We report a 48 -day old infant patient who weighed 3.8 kgs and was second order of birth. The patient was diagnosed with SVT at the fifth month of gestation. Postnatally, he spent two days in the NICU, where his sinus rhythm was restored via IV medications and then discharged on propranolol. Shortly thereafter, he developed recurrent tachycardia that was refractory to all available antiarrhythmics, including amiodarone and DC shock, for which he was referred to our center for evaluation.

**Figure 1.**
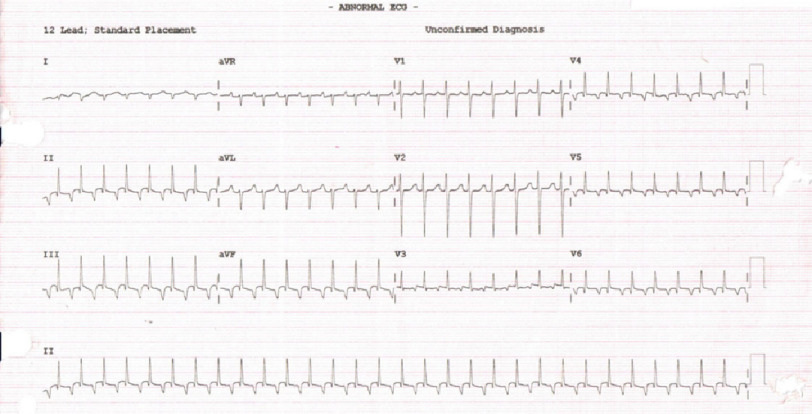
12 leads ECG during the tachycardia.

**Figure 2.**
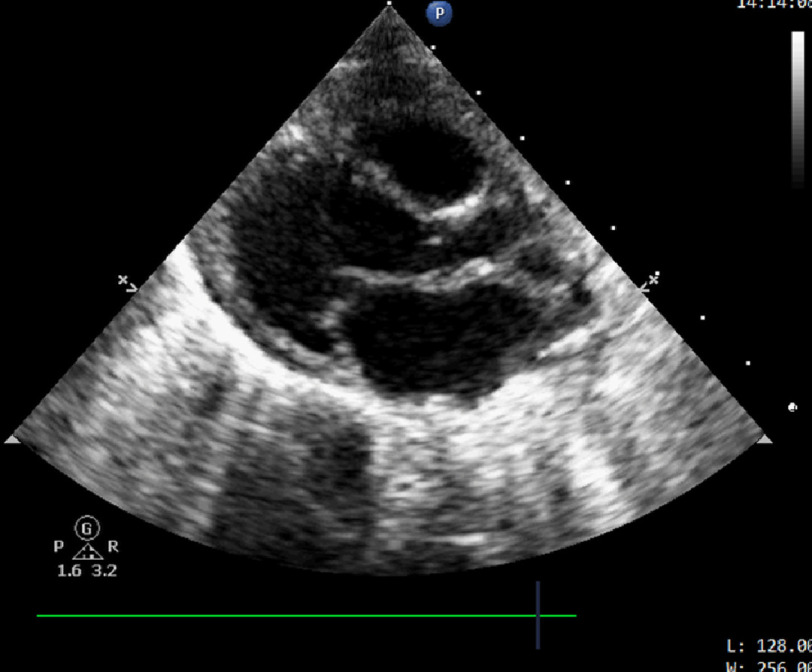
Transthoracic echo, parasternal long axis view shows dilated LV.

On presentation, the patient presented with incessant tachycardia (Figure 1) and stable blood pressure. However, echocardiography revealed a severely dilated left ventricle, with impaired systolic function and severe mitral regurgitation (Figure 2). After admission to PICU, several trials were performed to terminate or even control the tachycardia, with no success. A few hours later, the patient developed cardiogenic shock with severe hypotension and rising serum lactate levels, requiring intravenous inotropes and ventilatory support.

**Figure 3.**
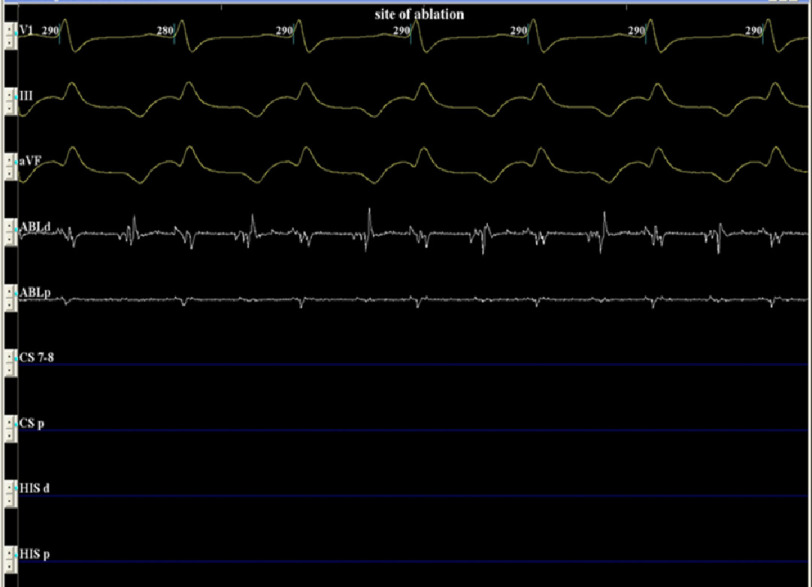
Intracardiac tracing shows the earliest atrial activation during the tachycardia.

**Figure 4.**
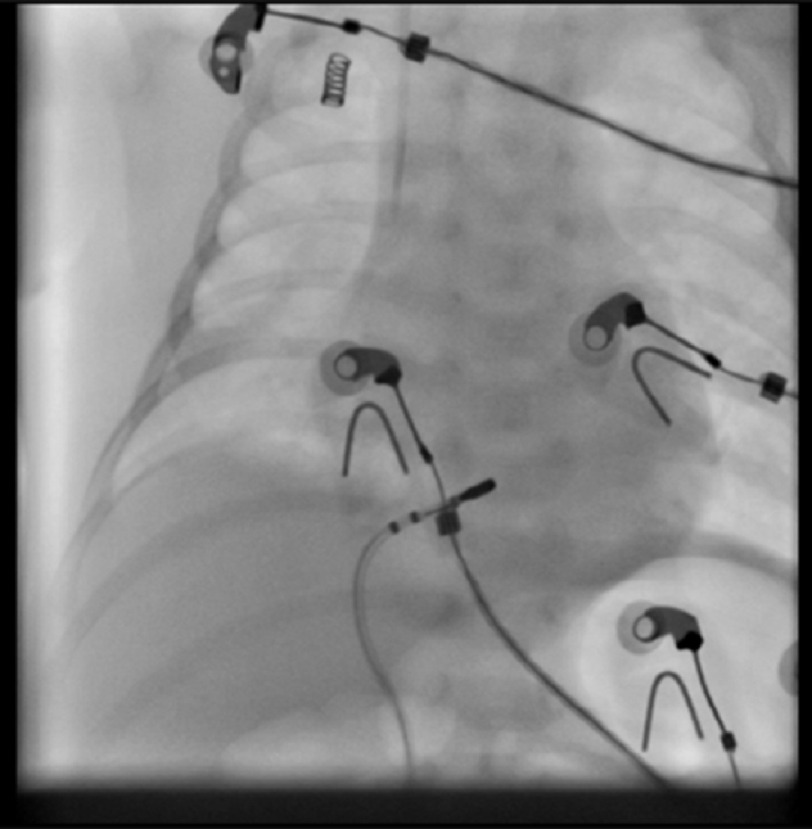
Fluoroscopy image shows 5F ablation catheter at the site of earliest activation.

**Figure 5.**
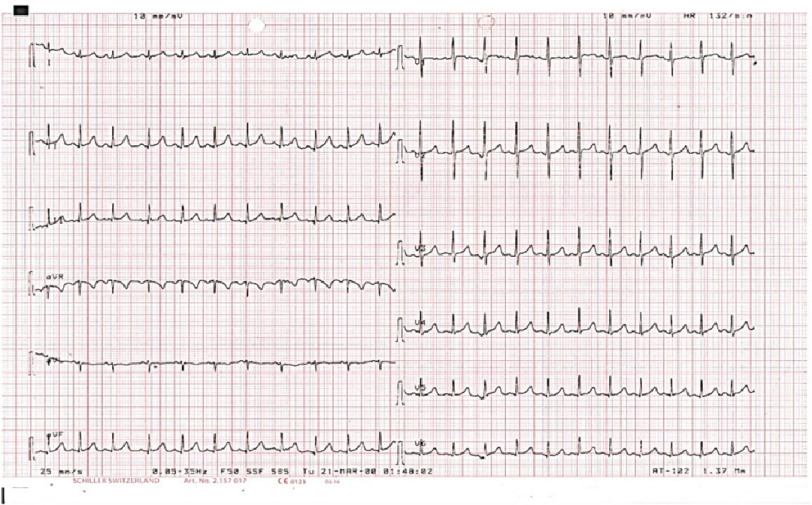
12 leads ECG post ablation illustrates sinus rhythm.

After team discussion and counselling of the parents, the patient was transferred to the Cath Lab for EP study and ablation. We obtained one difficult duplex-guided venous access and inserted a 5F sheath on the left side. A 5F 4 mm dry tip ablation catheter was used for mapping and ablation. After confirming the diagnosis of atrial tachycardia, a catheter was used for mapping to detect the earliest atrial activation in the ostium of the coronary sinus (Figures 3 and 4). Radiofrequency ablation was started at 20 watt with immediate termination of tachycardia with no recurrence even after the use of weight-adjusted doses of isoprenaline (Figure 5).

The patient had a smooth post-procedural course, with weaning of support within 3 days. He was committed to anti-failure treatment, including ACEI, BBs, and aldosterone antagonists, and was discharged home.

**Figure 6.**
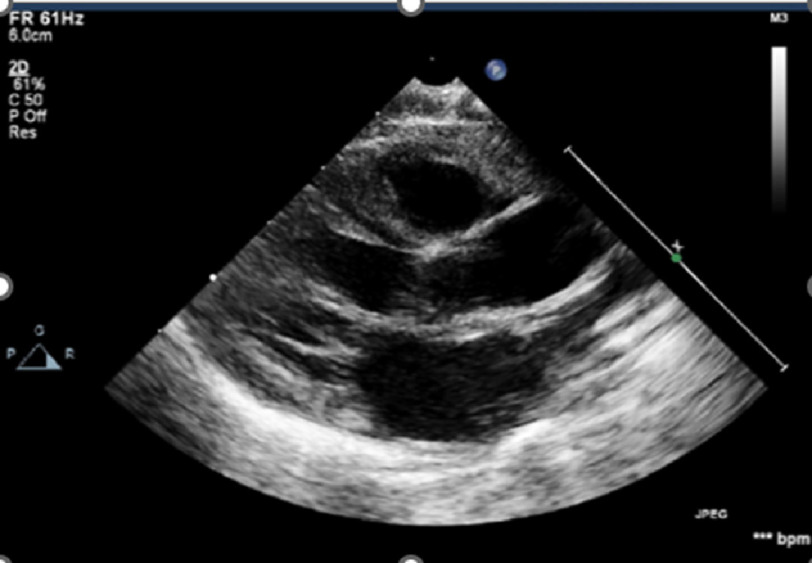
Transthoracic echo, parsternal long axis view shows recovered LV dimensions and function postablation.

During the follow-up visits, the patient had no recurrence of tachycardia, with gradual improvement of LV systolic function to be normalized three months later (Figure 6).

## Discussion

Supraventricular tachyarrhythmia is the most symptomatic pediatric arrhythmia resulting from an abnormal mechanism that requires cardiac structures above the bifurcation of the bundle of His for its continuation. Clinical symptoms of SVT are variable, ranging from asymptomatic or minor palpitations to more severe manifestations including heart failure^1^. In infancy, SVT predominantly results from accessory pathways and a small number of ectopic atrial tachycardias. In teenage life, there is a significant increase in the prevalence of atrioventricular nodal reentry tachycardia (AVNRT) particularly in females.

Ectopic atrial tachycardia is a rare cause of SVT in children accounting for 3.7–5.7% undergoing EPS. This mechanism typically involves centrifugal atrial activation away from a single source. In addition, multifocal atrial tachycardia (MAT)/chaotic atrial rhythm is well recognized and may have an ECG appearance with different P-wave morphologies or may be indistinguishable from AF.

The site of origin is variable, and includes the right and left atrial appendages, pulmonary vein ostia, and crista terminalis. Owing to its incessant nature, LV dysfunction, which is potentially severe in nature, is frequently observed.

In our case, all antiarrhythmic medications failed to control the tachycardia leading to deterioration of the haemodynamic state and LV systolic function, which is why ablation was mandatory, despite the fact that ablation at body weight less than 15 kg has been reported to have a higher rate of major complications when compared with older children. Special requirements should be available for ablation at such an age, and the presence of small-sized catheters, such as the 5F catheter, is highly needed.

TIC is defined as a myocardial dysfunction that is wholly or partially reversible after controlling for tachyarrhythmia. Despite a favorable clinical course for most patients, reports of sudden cardiac death, rapid deterioration in ventricular function with recurrence of tachyarrhythmia, and significant delays in reverse remodeling (“persistent negative remodeling”) despite successful arrhythmia therapy have been reported.

The goal of tachycardiomyopathy management is to achieve ventricular heart rate control or restore sinus rhythm. Options for restoration of sinus rhythm include electrical cardioversion, antiarrhythmic drugs, and catheter ablation of arrhythmia. Chronic or recurrent SVT associated with ventricular dysfunction is a class I indication for radiofrequency catheter ablation in pediatric patients.^2^

In TIC, previous small reports described weeks to months for functional recovery and years for reverse remodelling. The median recovery time in a larger study was <2 months in children.^3^

In our case, LV systolic function gradually improved over 3 months until full recovery.

## What have we learned?

• The diagnosis of TIC is difficult, and a high index of suspicion is required.
• Given the potential for recovery with appropriate treatment, a proactive approach, whether through rate or rhythm control, is recommended.
• Despite the risk of ablation in infants, it is sometimes mandatory, especially in those with tachycardia-induced cardiomyopathy after the failure of medical treatment.
